# The Electric Bath for Chilblains

**Published:** 1899-01-21

**Authors:** 


### THE ELECTRIC BATH FOR CHILBLAINS.

DR. LEWIS JONES,[1] of St. Bartholomew's Hospital, says that he has often been struck with the fact that children with infantile paralysis, who are very subject to chilblains in winter time, often become quite free from them during the electric bath treatment of their paralysis, and this has led him to try the effect of electricity in a case of chilblains of a severe type. Excellent results are stated to have followed. The method of application is very simple. An induction coil is used and the wires are attached to two metallic plates which are placed at the two ends of an ordinary earthenware foot-bath filled with warm water. The patient is instructed to use this bath at bedtime, for ten or fifteen minutes, whenever the slightest threatening of chilblains is noticed. The current is used as strong as it can be borne without discomfort, the effect being to make the feet warm with a glow which lasts until the patient goes to sleep. The same form of bath may also be used by those who habitually suffer from cold feet.

[1] Lancet, Jan. 14th.

